# Stimulatory Response of Celiac Disease Peripheral Blood Mononuclear Cells Induced by RNAi Wheat Lines Differing in Grain Protein Composition

**DOI:** 10.3390/nu11122933

**Published:** 2019-12-03

**Authors:** Susana Sánchez-León, María José Giménez, Isabel Comino, Carolina Sousa, Miguel Ángel López Casado, María Isabel Torres, Francisco Barro

**Affiliations:** 1 Departamento de Mejora Genética Vegetal, Instituto de Agricultura Sostenible, 14004 Córdoba, Spain; ssanchez@ias.csic.es (S.S.-L.); mjga06@ias.csic.es (M.J.G.); 2 Departamento de Microbiología y Parasitología, Facultad de Farmacia, Universidad de Sevilla, 41012 Sevilla, Spain; icomino@us.es (I.C.); csousa@us.es (C.S.); 3 Departamento de Gastroenterología Pediátrica, Hospital Virgen de las Nieves, 18014 Granada, Spain; miglcas@hotmail.com; 4 Departamento de Biología Experimental, Campus Universitario Las Lagunillas, 23071 Jaén, Spain; mtorres@ujaen.es

**Keywords:** PBMCs, RNAi, low-gluten wheat, celiac disease, NCWS

## Abstract

Wheat gluten proteins are responsible for the bread-making properties of the dough but also for triggering important gastrointestinal disorders. Celiac disease (CD) affects approximately 1% of the population in Western countries. The only treatment available is the strict avoidance of gluten in the diet. Interference RNA (RNAi) is an excellent approach for the down-regulation of genes coding for immunogenic proteins related to celiac disease, providing an alternative for the development of cereals suitable for CD patients. In the present work, we report a comparative study of the stimulatory capacity of seven low-gluten RNAi lines differing in grain gluten and non-gluten protein composition, relevant for CD and other gluten pathologies. Peripheral blood mononuclear cells (PBMCs) of 35 patients with active CD were included in this study to assess the stimulatory response induced by protein extracts from the RNAi lines. Analysis of the proliferative response and interferon-gamma (INF-γ) release of PBMCs demonstrated impaired stimulation in response to all RNAi lines. The lower response was provided by lines with a very low content of α- and γ-gliadins, and low or almost devoid of DQ2.5 and p31–43 α-gliadin epitopes. The non-gluten protein seems not to play a key role in PBMC stimulation.

## 1. Introduction

As basic staple foods, cereal grains substantially contribute to the dietary intake levels of energy, protein and fiber in the human diet. Wheat flour—containing approximately 9%–15% protein—is processed into a great variety of food products that humans consume on a daily basis, such as bread, cakes, noodles, biscuits, etc. Among the seed storage proteins (SSPs) of wheat grain, gluten is the most conspicuous, accounting for approximately 70%–80% of total protein. However, gluten is not a single protein, but a complex mixture of different proteins that accumulate during grain development, which ultimately provides wheat flour with unique physicochemical attributes which are a key factor in baking quality.

Wheat gluten features outstanding technological properties and nutritional benefits, but the intake of these proteins has also been related to triggering certain pathologies—of which, celiac disease (CD) is the best-known and most studied, affecting adults and children with an estimated global prevalence of approximately 1% [1,2]. In addition, interest has been focused on the non-gluten components of wheat—a group of proteins that include the amylase trypsin inhibitors (ATIs), which seem to activate the innate immune system [3].

In the case of CD, gluten is a strong environmental factor, but it also has a genetic and immunological component related to human leukocyte antigen (HLA)-DQ2 and HLA-DQ8 [4]. Due to their high content of proline and glutamine, gluten proteins—also known as “prolamins”—are resistant to their complete digestion in the human digestive tract. Thus, peptides produced as a result of the partial digestion of prolamins induce an autoimmune-mediated disorder that leads to small intestine inflammation, malabsorption, and villous atrophy in patients suffering from CD. A two-signal model is currently the most accepted model to explain CD [5]. According to this model, certain gluten peptides trigger an innate immune response, followed by a secondary antigen-specific adaptive response. The result of the innate response is the increase in permeability of the epithelial barrier, allowing gluten peptides to reach the lamina propria [6]. The most studied innate response activator peptide is known as p31–43, occurring in α-gliadins [7]. The autoimmune response is strongly enhanced as a consequence of the deamination of glutamine residues present in gluten peptides by tissue transglutaminase 2 (tTG2) in the intestinal mucosa. Deamidated peptides are capable of binding to HLA-DQ2 and HLA-DQ8 molecules present in antigen-presenting cells (APCs) [8], stimulating T cells that release pro-inflammatory cytokines such as IFN-γ, tumor necrosis factor-alpha (TNF-α) and interleukin-2 (IL-2). These cytokines damage enterocytes and produce intestinal lesions typical for CD [9]. Although several CD epitopes are found in the glutenin fraction of gluten, the majority of the immunogenic CD epitopes are found in the gliadin fraction of gluten [10]. Among the gliadins, the α-gliadins have the strongest immunogenicity, and the α-gliadin 33 mer is the main immunodominant toxic peptide in celiac patients. This peptide is present in the N-terminal repetitive region of α -gliadins and contains six overlapping copies of three different DQ2-restricted T cell epitopes with highly stimulatory properties [11].

The only treatment available for CD patients is a lifelong strict gluten-free diet. However, completely avoiding gluten in the diet is arduous, as these proteins are widely used in the food industry and are added to many foodstuffs—some of which are naturally lacking gluten Further, gluten-free products tend to be less healthy than those containing gluten, as high amounts of fat and sugar are involved in their production, with the aim of providing gluten-free (GF) products with a texture that mimics the viscoelastic properties of gluten proteins [12]. For this reason, the development of wheat varieties with reduced immunogenic profiles should be considered an excellent ingredient to improve the diet of patients with CD, as well as for those with Non-Celiac Gluten Sensitivity (NCGS). In addition, these wheat varieties could undoubtedly also apply to the general population, in particular for those who, for whatever reasons, want to reduce the intake of gluten. In fact, in a recent study, the beneficial effect of a low-gluten diet was reported in comparison to high-gluten diets [13]. Results showed that a low-gluten diet changed the gut microbiome of participants, reduced their gastrointestinal discomfort, and resulted in a small weight loss.

One promising approach to reduce gluten content and immunogenicity is the down-regulation of immunodominant peptides by interference RNA (RNAi) [14,15]. In previous works, we implemented this technology to reduce the expression of specific gliadin fractions [16] as well as all three gliadin fractions [17,18] in bread wheat. Protein extracts from RNAi lines with the three gliadin fractions regulated downwards showed a pronounced reduction in T cell response when tested in vitro [18]. However, the down-regulation of both specific and all gliadin fractions provided a compensatory effect with other protein fractions [19,20]. This is particularly important when non-gluten proteins (NGPs) are used for this compensation as metabolic proteins and chloroform/methanol soluble proteins (CM-like), such as the α-amylase/trypsin inhibitor family, β-amylase and serpins, were related to wheat allergens [21].

The present work was designed to study the in vitro response of peripheral blood mononuclear cells (PBMCs) from CD patients to different RNAi lines differing in gluten and NGP composition. Modifications in wheat grain protein composition by RNAi led to a reduction in the CD-related epitopes of the most immunogenic fractions. The findings of this research may be useful to establish an optimal protein composition to pursue the maximum reduction in the immunogenic potential for CD for the development of new wheat varieties.

## 2. Materials and Methods

### 2.1. Plant Material

Seven RNAi lines derived from bread wheat cv. Bobwhite (BW208) and their corresponding wild-type lines were used in this study (Table 1). The RNAi lines and transformation vectors used were previously reported [17,18,20,22]. Lines were obtained by using combinations of different RNAi fragments designed to target different gliadin fractions—the ω-, α- and γ-gliadin and low-molecular-weight (LMW) fraction of glutenins. Each silencing fragment was expressed under a D-hordein endosperm-specific promoter [23].

### 2.2. Reversed-Phase High-Performance Liquid Chromatography (RP-HPLC) Quantification of SSP Proteins

Gliadins and glutenins from 100 mg of flour from three biological replicates were extracted stepwise. Both fractions were quantified by RP-HPLC following the protocol reported by Piston et al. (2011) [22].

### 2.3. Total Protein and NGP Quantification

The protein content of whole flour was calculated from the Kjeldahl nitrogen content (%N × 5.7) according to the standard International Association for Cereal Chemistry (ICC) method no. 105/2 (ICC, 1994). NGPs, expressed in percentage of dried weight (% DW), were calculated as follows:

(Total protein in % − (Prolamin content in µg/mg × 10)/(100 − moisture in %)).(1)

### 2.4. Liquid Chromatography–Tandem Mass Spectrometry Analysis

Total protein extraction and pepsin and trypsin digestion of samples were carried out from 2 g of flour by following a previously reported protocol [17], with two exceptions: a greater ratio of flour/extraction volume was employed, and centrifuge filtration of extracts (Corning 45 µm Nylon tubes) prior to enzymatic digestion was performed. Protein digests (1.5 µg) were analyzed after being cleaned with a SEP-PAK C18 cartridge (Waters, Milford, MA, USA) by one-dimensional nanoscale liquid chromatography–tandem mass spectrometry (LC–MS/MS) on an Eksigent NanoLC-1D plus (AB SCIEX) coupled to a 5600 Triple TOF^®^ mass spectrometer (AB SCIEX) equipped with an Acclaim PepMap 100, 100 μm × 2 cm (Thermo Fisher Scientific, Waltham, MA, USA) precolumn and NanoACQUITY UPLC 1.7 μm BEH130 C18, 75 μm × 150 mm (Waters) HPLC column. For the proteomics analysis, resulting data were searched against the National Center for Biotechnology Information (NCBI) protein database for *Triticum aestivum* species without any enzyme restriction. Searches were conducted using Mascot Server 2.4 (Matrix Science, London, UK). Only peptides with scores higher than 20 were extracted for further analyses. CD epitope content was determined using BlastP to search against the CD epitopes described by Sollid et al. [24] and the α-gliadin peptide 31–43 (Table S1) in peptides longer than eight amino acids identified by LC–MS/MS analysis.

### 2.5. Gluten Content Determination by Competitive Enzyme-Linked Immunosorbent Assay (ELISA)

The gluten content of whole flour was measured by G12 monoclonal antibody (moAb) as described previously [17]. Each sample was measured in triplicate. Results were expressed in parts per million (ppm) in dry matter.

### 2.6. Peripheral Blood Mononuclear Cell Proliferation and interferon (IFN)-γ Production Analysis

Patients with active CD on a gluten-containing diet (*n* = 35) were included in this study. The diagnosis of CD was primarily determined by serological screening tests and finally confirmed with biopsy of the small intestine. The mucosal specimens were graded independently according to the Marsh–Oberhuber classification [25,26]. Subjects were prospectively screened for CD using antiendomysial antibodies (AAEMs), anti-tissue transglutaminase antibodies (AATGs), and CD-specific human leukocyte antigen (HLA) typing (Table S2). The local Ethics Committee of the Hospital “Virgen de las Nieves” (Granada, Spain) approved the study protocol. Written consent was obtained from parents or legal guardians of children involved.

PBMCs were isolated from 6 mL of heparinized blood by Histopaque gradient centrifugation (Sigma Aldrich, Madrid, Spain) and cultured at a density of 1 × 10^6^ cells/mL in RPMI-1640 culture medium (Gibco, Thermo Scientific, Madrid, Spain) supplemented with 10% fetal bovine serum (Gibco, Thermo Scientific, Madrid, Spain), 1% penicillin-streptomycin, and 0.1% gentamicin (Sigma-Aldrich).

The above described PT-digested protein extracts were also used to study immunogenic potential by PBMC proliferation assay and IFN-γ release. Rice flour and synthetic extract of 33 mer peptide were used as the negative and positive controls, respectively. After 48 h of culture, PBMCs were incubated with 50 µg/mL of protein extracts from the different lines and controls (33 mer peptide, rice and blank without protein extracts added). Each experiment was carried out in duplicate. Cultures were collected after 24 h of stimulation, separating PBMCs for cell proliferation studies and supernatants for IFN-γ analysis. Supernatants from the PBMC culture were stored at −80 °C until IFN-γ determination was carried out using a commercial ELISA kit (Thermo Scientific, Madrid, Spain) in accordance with the manufacturer’s instructions. Standards were run on each plate. Assay sensitivity was less than 2 pg/mL.

Cell proliferation was determined by the ELISA 5-bromo-2-deoxyuridine (BrdU) cell proliferation test (Millipore Chemicon, CA, USA). Proliferative responses of PBMCs were defined as a stimulatory index (SI)—this variable represents the specific proliferation of a sample as the mean absorbance at 450 nm after stimulation divided by the mean absorbance of PBMCs exposed to the culture medium alone.

### 2.7. Statistics

Statistical software R version 3.5.1 (Ihaka and Gentleman, 1996) was used for data analysis and some plots. Analysis of variance (ANOVA) followed by the two-tailed Dunnett test for mean multiple comparisons was used for establishing differences between lines. Normal distribution and homogeneity of variance were previously tested by the Shapiro–Wilk normality test and the Levene test, respectively. Pearson’s R was used to determine data correlation. Figures were drawn using the Microsoft Excel and PowerPoint software (Microsoft Corporation). The libraries FactoMineR and Factoextra were used for Principal Component Analysis (PCA) analysis and graphical output, respectively.

For cell proliferation and IFN-γ assays, each experiment was carried out in duplicate on separate days. Resulting data are expressed as the mean and SD. All statistical analyses were performed with the STATGRAPHICS Centurion XVI program. Analysis of variance (ANOVA) was used, followed by the Tukey test for mean multiple comparison.

In this study, *P* values lower than 0.05 were considered significant.

## 3. Results

### 3.1. Grain Protein Composition of RNAi Lines

**Figure 1:**
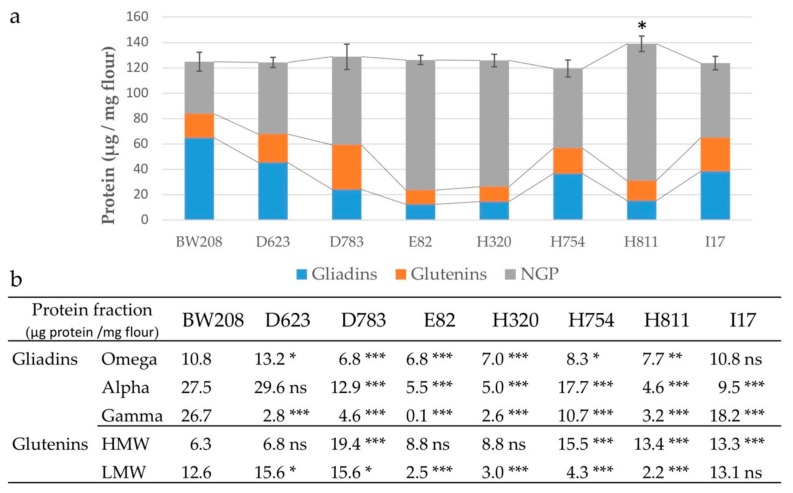
Grain protein composition of RNAi lines and wild-type (BW208) lines: (**a**) Protein distribution of three main protein groups in the wheat grain; (**b**) Protein distribution of prolamin fractions. NGPs, non-gluten proteins; HMW, high-molecular-weight glutenin subunits; LMW, low-molecular-weight glutenin subunits. Dunnett multiple comparison of means with BW208 wild-type line; ns = not significant; * *p* < 0.05; ** *p* < 0.01; *** *p* < 0.001.

The content of the different gluten and NGP fractions comprised in wild-type BW208 and the RNAi lines are shown in Figure 1. All RNAi lines present the reduction in the gliadin fraction, while the range of variation in glutenins depends on the line analyzed. The strongest reduction for gliadins corresponds to lines E82, H320, and H811 (Figure 1a)—all three of which contain a combination of two plasmids (Table 1). Total glutenins of lines D623, H754 and H811 do not present significant change with respect to the BW208 wild-type line. Conversely, an increase in the glutenin fraction occurs in lines D783 and I17, in contrast to the reduction observed on the glutenin content of lines E82 and H320. As shown in Figure 1a, the NGP fraction is up-regulated for all RNAi lines, and particularly for lines E82, H320, and H811. The reduction in the gliadin fraction in the grains is compensated through an overexpression of other proteins, so that the total protein content remains unaltered for all lines except for line H811—in which, the total protein content is significantly affected in comparison to that of the BW208 wild-type line.

Concerning the gluten fractions, which include gliadins and glutenins (Figure 1b), all RNAi lines show significant changes in some of the protein fractions in comparison to the BW208 wild-type line, regardless of the fragment used for silencing. Lines D623, H754 and I17 hold RNAi vectors aimed to silence a single gliadin fraction (γ-, ω- and α-gliadins, respectively), but D623 is the only line showing a reduction only on its specific target (Figure 1b). In addition to the resulting down-regulation of their specific targets, line I17, targeting α-gliadins, also presents a lower amount of γ-gliadins, and line H754, targeting ω-gliadins, also showed a decrease in α- and γ-gliadin fractions. E82 is the line with the highest reduction in gliadins, followed by lines H320 and H811, both with α- and ω-gliadins as targets, but with strong reduction in the γ-gliadin fraction. Line D783 shares one construct with line E82, but the latest has an additional construct to more precisely target the γ-gliadin fraction. In comparison to D783, the E82 line has a strong reduction in γ- and α-gliadins, suggesting a synergic effect when more than one gliadin fraction is used as a silencing target.

Within the glutenins, the HMW fraction is significantly up-regulated in comparison to that of the BW208 wild type in lines D783, H754, H811 and I17, and not significantly affected in the other three RNAi lines. HMW glutenins are not included as targets in any of the RNAi vectors and do not present down-regulation in any of the RNAi lines. However, probably due to the grain protein compensation system, there is an overexpression of HMW in most lines, particularly in line D783, with up to a 3-fold increment. In contrast, the LMW fraction is significantly affected in all but one RNAi line; in lines D623 and D783, the LMW fraction is up-regulated, while in lines E82, H320, H754, and H811, LMW fraction is strongly down-regulated (Figure 1b).

Overall, E82, H320 and H811 are the lines with a higher reduction in prolamins, the differences among them being in the stronger reduction in γ-gliadins for E82, and the stronger up-regulation of HMW in line H811.

### 3.2. Analysis of Pepsin and Trypsin Protein Extract Digestion (PT-Digestion)

**Figure 2:**
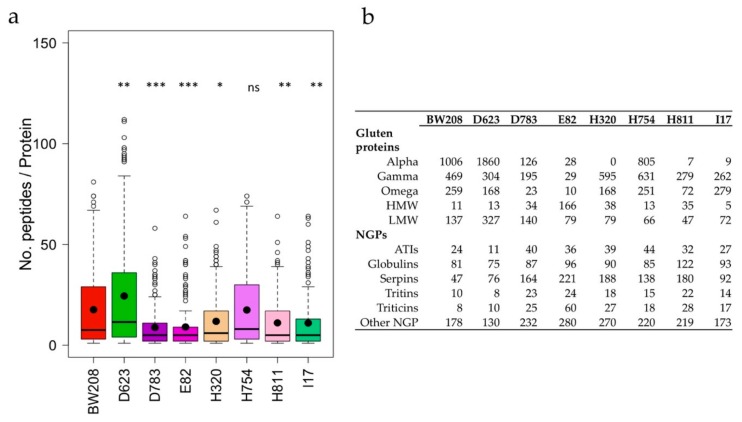
(**a**) Average number of peptides per protein and genotype identified by liquid chromatography–tandem mass spectrometry (LC–MS/MS). (**b**) Number of protein peptides identified corresponding to the different gluten and non-gluten protein fractions. NGPs, non-gluten proteins; ATIs, amylase trypsin inhibitors; HMW, high-molecular-weight glutenin subunits; LMW, low-molecular-weight glutenin subunits. Dunnett multiple comparison of means with BW208 wild-type line; ns = not significant; * *p* < 0.05; ** *p* < 0.01; *** *p* < 0.001.

Total protein was extracted from flour of the six RNAi wheat lines and the BW208 wild-type line and then subjected to pepsin and trypsin digestion (PT-digestion). Resulting fragments were analyzed by LC–MS/MS and identified through a *Triticum aestivum* species restricted search in the National Center for Biotechnology Information (NCBI) database. The silencing of specific prolamin fractions by RNAi resulted in significant differences in the number of peptides per protein identified in PT-digested flour for all RNAi lines except H754 (Figure 2a). Line D623 showed a higher number of peptides per protein than the BW208 wild-type line, whereas all other lines but H754 provided a significant lower number of peptides per protein. A variation in the number of peptides per protein proves a variation on protein composition after silencing by RNAi. In this regard, lines H320, H811 and I17 showed the lowest number of α-gliadin peptides in comparison to the BW208 wild-type line (Figure 2b). In contrast, line D623 had an important increase in α-gliadin peptides. In general, lines E82, D783, and H811 showed a reduction in the peptides of all three gliadin fractions. Compensatory effects described above for HPLC data are also reflected in the number of peptides per protein in the glutenin and in the NGP fractions. For example, the number of identified peptides corresponding to ATIs, globulins, serpins, triticins, and other NGPs are notably increased in some RNAi lines (Figure 2b). Particularly interesting is the increment in serpin peptides for all RNAi lines.

**Figure 3:**
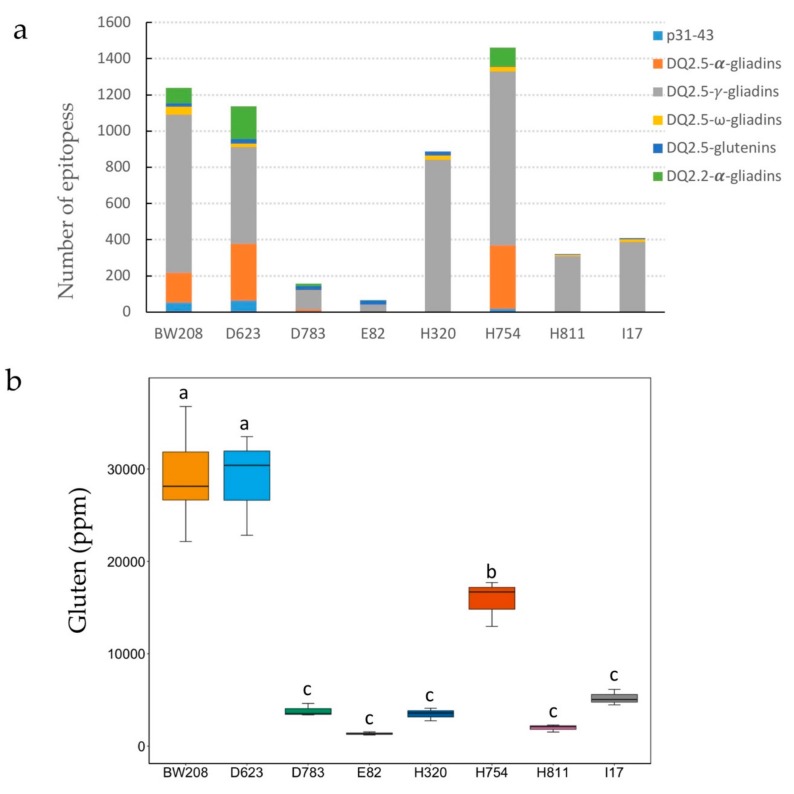
(**a**) Number of DQ-restricted epitopes identified in LC–MS/MS peptides. (**b**) The gluten content — based on monoclonal antibody G12 — was measured by an enzyme-linked immunosorbent assay (ELISA) and expressed in parts per million (ppm). Lines with the same letter are not significant different according to the Tukey test comparison of means (*p* < 0.05).

Peptides were searched for the presence of DQ-restricted epitopes, related with adaptive immune response, and for the p31–43 peptide ocurring in α-gliadins, which is linked with innate immune activation (Table S1). Results of the analysis showed a notable decrease in the number of CD epitopes in four of the RNAi lines (Figure 3a). There is only one line (H754) that presents a higher number of total epitopes in comparison with the wild-type line. From the epitopes identified in the wild-type line, the vast majority corresponds to γ- and α-gliadins epitopes. The p31–43 fragment hits were reduced in all RNAi lines except in D623, and they were not found in I17, H320 and H811. The γ-gliadin-related epitopes are the most abundant in all samples analyzed including the wild-type line. The reduction in the number of epitopes found in this gliadin fraction ranges from 4% in line H320 to 95% in E82 when compared with the wild-type line. Epitopes corresponding with α-gliadins were not identified in peptides from three of the RNAi lines (I17, E82 and H320) and were reduced by over 93% in lines D783 and H811. In contrast, they appeared notably increased in lines D623 and H754, where they are overexpressed. Epitopes present in ω-gliadins are also reduced in all lines, with an average reduction of over 80% in lines containing inverted repeated (IR) fragments for this target and absent in line E82. Regarding glutenin epitopes, half of the lines (D623, D783, E82 and H320) had a higher content of these epitopes. Whereas, the other three lines (H754, H811 and I17) present a decrease in the number of epitopes for this protein fraction (Figure 3a). Results from the proteomic analysis showed that lines with a greater reduction in the total number of immunogenic CD epitopes are E82, D783, and I17, with an average reduction of 94%, 88%, and 62%, respectively, compared with the wild-type line. It is noteworthy that line E82 is completely devoid of CD epitopes from the highly immunogenic α- and ω-gliadins and contains only 5% of γ-gliadin epitopes.

### 3.3. Gluten Immunogenicity by G12 moAb

Gluten content in parts per million (ppm) of flour from all lines was determined by a competitive ELISA system with G12 moAb [27]. As shown, gluten content (ppm) was strongly reduced in five of the seven lines compared with the wild-type line (Figure 3b), with an average reduction of 85% and a maximum reduction of 95% corresponding to line E82. RNAi line D623 showed comparative gluten content to that of the wild-type line, while line H754 had a significant lower value in comparison to the wild-type line. Overall, lines E82 followed by H811 are the ones with lower immunogenic potential. The level of reduction in G12 values was strongly correlated with the content of α-gliadins (*R* = 0.972; *p* < 0.001), total gliadins (*R* = 0.888; *p* = 0.003), and total prolamin (*R* = 0.772; *p* = 0.025) fractions, as determined by RP-HPLC. In addition, G12 also provided significant correlations with the number of DQ2.5 from α-gliadins (*R* = 0.845; *p* = 0.007) and p31–43 (*R* = 0.973; *p* < 0.001) peptides, present in α-gliadins, and with the total number of gliadin peptides (*R* = 0.729; *p* = 0.041).

### 3.4. Stimulatory Response of PBMCs from CD Patients

**Figure 4:**
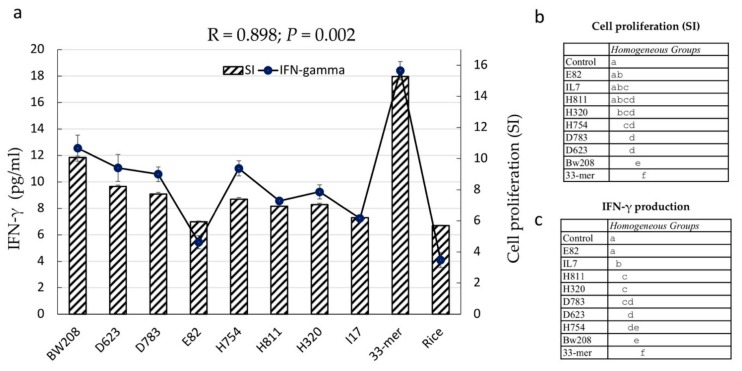
(**a**) Immunogenicity of different RNAi wheat lines. Bars represent the proliferative responses of peripheral blood mononuclear cells (PBMCs) to pepsin and trypsin digested (PT-digested) protein extracts from the wild-type (BW208) and RNAi wheat lines defined as a stimulatory index (SI). Line and dots represent IFN-γ release by PBMCs in response to PT-digested protein extracts from the wild-type (BW208) and RNAi wheat lines. Results represent the mean of 35 patients ± standard deviation (SD). (**b**) Cell proliferation and (**c**) IFN-γ production groups according to Tukey HSD multiple range test. Different letters denote significant differences (*p* < 0.05).

**Figure 5:**
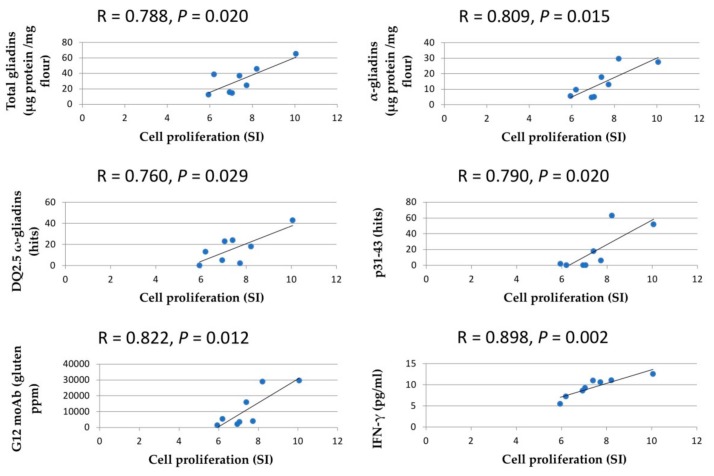
Pearson’s correlation analysis of variables measured by Reverse-Phase High-Performance Liquid Chromatography (RP-HPLC), proteomics, PBMCs and G12 moAb.

Cell proliferation assay and IFN-γ release were carried out using the PT-digested flour from the RNAi lines in order to determine their ability to activate the stimulatory response of PBMCs from CD patients (Figure 4). Immunogenic potential was tested as a measure of the capacity to trigger cellular proliferation (Stimulation Index, SI) in cultures from 35 different child patients suffering from celiac disease and under a gluten-containing diet (Table S2), using BW208 wild-type flour and 33 mer peptide as the positive controls, and PT-digested rice flour as the negative control. Further, a control without protein extracts added in the culture cells (blank group) was included for reference values to compare the effect of peptides in cells of patients with CD, under the same conditions of cell culture. There is an excellent correlation between cell proliferation and IFN-γ release (*R* = 0.898; *p* = 0.002) (Figure 5). As expected, the 33 mer peptide and rice negative controls showed the maximum and minimum, respectively, of both cell proliferation and IFN-γ release. BW208 was the wheat line with higher cell proliferation and IFN-γ values (Figure 4). As shown, changes observed in the grain prolamin fractions, as a consequence of RNAi silencing, led to changes in the cell proliferation and IFN-γ release of RNAi lines. All RNAi lines have cell proliferation values (SI) lower than the BW208 wild-type line, showing an average cell proliferation reduction of approximately 30%. A reduction in the proliferative response of PBMCs was seen in lines E82, I17 and H811, with SI values similar to that of the rice negative control (Figure 4, Table S3). In contrast, lines BW208, D623, D783, H320 and H754 showed significant higher values than the rice negative control. IFN-γ release confirmed the impaired stimulatory capacity of PT-digested flour from the seven RNAi lines (Figure 4), with RNAi lines showing an average reduction of 28% with respect to the BW208 wild-type line. Lines E82 and I17 provided the lowest values and, in the case of E82, this was not significantly different to that of the rice negative control (Figure 4, Table S4), demonstrating a decrease in the immunotoxicity of flour by the reduction in certain gluten fractions by RNAi.

Cell proliferation was positively correlated with both the α-gliadin (*R* = 0.0809; *p* = 0.014) and total gliadin (*R* = 0.788; *p* = 0.020) contents as determined by HPLC (Figure 5). In addition, cell proliferation showed significant correlation with gluten content provided by G12 moAb (*R* = 0.822; *p* = 0.012) and with the number of p31–43 (*R* = 0.7902; *p* = 0.019) and DQ2.5 ω-gliadin (*R* = 0.760; *p* = 0.028) epitopes detected in mass spectrometry peptides (Figure 5).

Our data confirm notable differences between the RNAi lines for cell proliferation and IFN-γ release. Overall, the RNAi lines E82 and 17 were highly inefficient in stimulating the immunogenic response by cell proliferation and IFN-γ release. Both lines do not differ significantly from the rice negative control (Figure 4, Tables S3 and S4), indicating that the reduction in the gliadin fractions in these lines, together with the reduction in the LMW and the increment in HMW and NGP fractions, results in a significant decrease in the immunogenic potential of their protein extracts.

**Figure 6:**
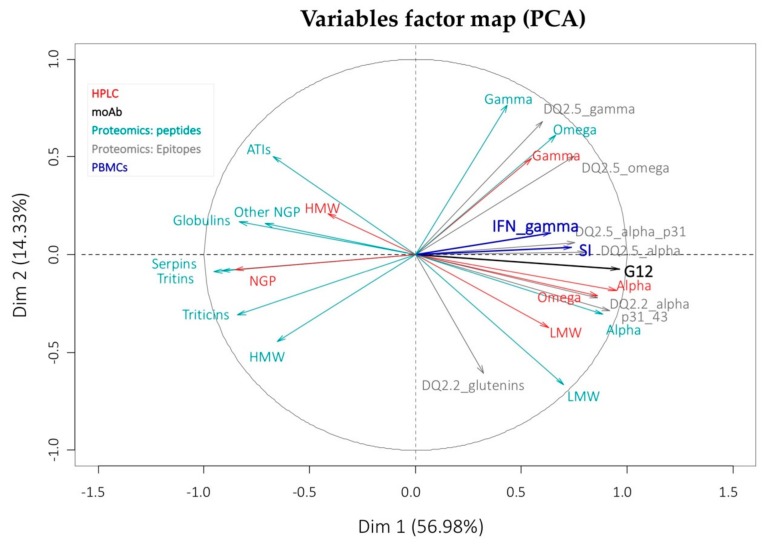
PCA analysis of the variables measured by RP-HPLC, proteomics, PBMCs and G12 moAb.

The relationships described above among all the parameters tested (quantification by HPLC of protein fractions, the results of the proteomics analysis and the proliferation of PMBCs) are well reflected in the PCA analysis (Figure 6). The first two dimensions represent 80% of the variation in the set of variables in the seven lines studied and show that all variables related to immunogenicity have a positive value in the first axis; that is, they vary in the same direction as the proliferation values of PBMCs and IFN-γ. These last two have an almost null value for the third dimension, which represents 9% of the variation, and this implies that its variation is well explained only with the first two axes. All variables related to NGPs and HMW glutenins vary in the opposite direction on the first axis, suggesting that they do not contribute to the immunotoxicity of the lines for celiac patients.

## 4. Discussion

RNAi technology has demonstrated to be a powerful approach for the down-regulation of different gluten genes encoding the proteins responsible for CD and other gluten-related pathologies. RNAi was used to down-regulate the expression of specific gliadin genes—γ-, ω-, and α-gliadins [16,17,28]—and even gliadin genes from more than one family at once [17,18,29]. The proteome of these lines was studied in detail and showed a compensatory mechanism to fill the gap left by the silenced gliadins [19,28]. This compensatory mechanism also operates on other glutenins and NGPs and, therefore, glutenins (HMW and LMW) and NGPs such as serpins, ATIs, globulins, or triticins are increased in addition to the silencing of gliadins. Therefore, RNAi down-regulation has a direct effect on the composition and proportion of the gluten and non-gluten fractions. In this work, we report a comparative study of the stimulatory response of RNAi lines, targeted at different gliadin or glutenin fractions, and differing in grain protein composition relevant for CD and other gluten pathologies.

This set of RNAi lines (Table 1) was produced by the expression of one or two RNAi silencing fragments under the control of a D-hordein endosperm-specific promoter. Consequently, specific gliadin fractions, as well as all three gliadin families, were strongly down-regulated. All characteristics of these RNAi lines are summarized in Table S5. Although the down-regulation of specific gliadins is observed, a stronger down-regulation of the targeted gliadin groups is provided when RNAi constructs targeting different groups work together in the same line. This pleiotropic effect is clear in lines E82 and D783, which share one construct targeting all gliadins groups. However, line E82 also includes a construct (pghpg8.1) designed to specifically target the γ-gliadins, and E82 presents a higher reduction in the γ-gliadins than D783. The specificity of pghpg8.1 is reflected in line D623, where silencing only occurs in γ-gliadins (Figure 1b). This fact suggests a synergic effect when more than one gliadin sequence is used as a silencing target. This also occurs in lines H811 and I17, as both contain a construct directed to α-gliadins, but this group of proteins was more reduced in line H811 (Figure 1b), which has an additional construct also targeting ω-gliadins (Table 1).

The silencing of specific gluten-protein fractions by RNAi provided a compensatory mechanism with other proteins, which means that the total protein content does not present significant changes in the RNAi lines in comparison to the wild-type line, with the exception of line H811. This compensatory mechanism mainly involves NGP and HMW glutenins. The latter are major determinants of bread-making quality and the higher proportion of this fraction, present in some lines, could indicate reasonable baking quality. Gil-Humanes et al. [30] reported lines with increased HMW as a consequence of RNAi silencing, providing flours with increased stability and better tolerance to over-mixing. In agreement with this, Altenbach et al. [28] reported an increment in the HMW subunits when ω-1,2 gliadin genes were down-regulated by RNAi, leading to an increment in the mixing time and tolerance. The up-regulation of NGPs occurs in all lines independently of the gluten fraction silenced. The increment in NGPs, particularly triticins [31], led to wheat lines with improved nutritional properties, since its lysine content is significantly higher than that of normal flour [30]. The NGPs include metabolic and structural proteins, such as CM-like and serpin proteins, which have been not only related to wheat allergy [32,33] but also reported as novel target antigens in CD humoral response [34]. The compensatory mechanism was corroborated with the analysis of PT-digested proteins from the flour of RNAi lines. An increment in the number of glutenin and NGP peptides was found in the RNAi lines. This increment is particularly important for ATIs, serpins and triticins, which corroborate previous observations using two-dimensional (2D) gel electrophoresis and LC–MS/MS analysis [19,28].

The gliadin fraction of gluten seems to play a major role in CD, as the most immunogenic epitopes are on these proline- and glutamine-rich proteins [10]. PT-digested peptides from RNAi lines were searched for the presence of relevant T cell epitopes recognized by cluster of differentiation 4 (CD4)+ T cells and binding to human leukocyte antigens (HLA)-DQ. DQ2.5-restricted epitopes have a higher risk for CD [24], because of the ability of HLA-DQ2.5 molecules to form stable complexes with a large gluten peptide repertoire [35]. DQ2.5 epitopes encoded by α-gliadins were not found in three of the RNAi lines, while those DQ2.5 epitopes encoded by γ-gliadins were strongly reduced in four lines. However, DQ2.2 and p31–43 α-gliadin epitopes were found in line E82 at a very low frequency. Lines devoid of α-gliadin epitopes still contain α-gliadin proteins as determined by RP-HPLC (Figure 1b) and corroborated by mass spectrometry analysis (Figure 2b). As reported by Ozuna et al. [36], there is a significant number of α-gliadin sequences devoid of immunogenic epitopes, and this could in part explain the observed discrepancy between RP-HPLC and the number of epitopes detected by mass spectrometry. On the other hand, the gliadin family is a complex mix of proteins and the exact number of genes encoding those proteins is variable between genotypes and not yet well known. Therefore, this is an additional limitation of protein databases, as not all protein sequences and peptides are deposited in them. Despite the mentioned limitations, there was an excellent correlation between the α-gliadins determined by RP-HPLC and the number of α -gliadin peptides determined by mass spectrometry (*R* = 0.841; *p* = 0.009). Moreover, results by G12 were also correlated with both the content of α-gliadins as determined by RP-HPLC (*R* = 0.972; *p* < 0.001) and the number of α-gliadin epitopes as determined by mass spectrometry (*R* = 0.845; *p* = 0.008).

All lines with the exception of D623 have a significant reduction in the gluten content detected by the G12 moAb. This antibody was developed against the 33 mer peptide, present in the α -gliadin fraction of wheat flour and recognized as the most immunogenic peptide present in wheat [37], as it contains six copies of two overlapping T cell epitopes—three copies of DQ2.5-glia-α1 and three copies of DQ2.5-glia-α2. Moreover, G12 recognize other gluten immunogenic peptides resistant to intraluminal and serum proteases that are recognized by the T cells of patients with CD [38,39,40]. G12 immunodepletion experiments with hydrolyzed gluten showed that this antibody reacted with those with the highest immunoactivity for celiac patients [41]. Although lines E82 and H811 showed the lowest values for immunogenic gluten, there is a group of five RNAi lines with no significant differences between them for G12 moAb values.

The PBMC proliferative response and release of pro-inflammatory cytokine INF-γ after exposure to flour was assayed in the seven RNAi lines and compared to the BW208 wild-type line and 33 mer peptide as positive controls, and to rice protein extracts as negative controls. PBMCs, circulating immune cells mainly constituted by T lymphocytes and B lymphocytes, play a key role in the inflammatory system. Their response and cytokine release is sensitive and specific to the presence of gluten antigenic proteins and peptides in CD patients [42]. As described above, the RNAi lines used in this study showed contrasting grain protein composition related to CD and other gluten pathologies. Protein extracts from two lines used in this study (E82 and D783) were previously assayed in vitro [18], showing a pronounced reduction in the proliferative responses of gliadin-specific T cell clones. However, the use of T cell clones allows the identification of variation in the stimulation by specific peptides but does not quantify their contribution to the overall immune response to gluten in patients suffering from CD. PBMC proliferation results and IFN-γ release reported in this study corroborate the overall immunogenicity reduction in these two lines.

PBMC proliferation assay results showed that the protein extracts from the seven RNAi lines have impaired ability to activate the stimulatory response of PBMCs from CD patients, corroborating a reduction in those gluten proteins that are able to trigger CD immune response. Differences in protein composition also revealed differences in CD epitope composition. Proteomic data showed that RNAi lines tested here are not completely devoid of CD stimulatory peptides, as they still contain small amounts of gliadins as well as glutenin proteins. However, not all CD epitopes are equally immunogenic [37], and our PBMC results agree with this. The α-gliadin fraction of gluten is known to contain the most immunogenic CD epitopes, including the 33 mer peptide, which is highly resistant to digestion. Lines E82, I17 and H811 contain very low or no DQ2.5 and p31–43 α-gliadin epitopes, as well as a very low content of gluten as determined by G12, and they showed the lowest capacity of inducing PBMC proliferation, presenting no significant differences in comparison with the rice control. Lines H320 and I17 also contain comparable levels of gluten and very low or no DQ2.5 and p31–43 α-gliadin epitopes, and both differ in PBMC response and IFN-γ release. However, both lines are different in DQ2.5 γ-gliadin epitope composition.

Although gliadins from wheat seem to be the primary trigger of PBMC proliferation and IFN-γ release, other components of the protein profile such as metabolic proteins called α-amylase/trypsin inhibitors (ATIs) have been identified as important factors for the development of symptoms [21]. Nevertheless, RNAi lines where NGPs are highly increased have a lower SI, suggesting that these proteins may have a minor contribution in activating the proliferative response of CD PBMCs.

Proteomic and PBMC analysis agree that lines H811, I17, and particularly E82 have the lowest immunogenic potential. Line E82 presents the lowest amount of prolamins as determined by HPLC, with a reduction of 99%, 80% and 37% of γ-, α- and ω-gliadins, respectively. Therefore, comparing the variation in the protein profile, together with the reduction in the immunogenic potential of these lines, it can be concluded that line E82 is of great interest for obtaining foodstuff with reduced toxicity. In addition, the reduction in gliadins in this line is not compensated with an increase in the glutenin fraction but with NGPs, suggesting a minor role of these proteins on the stimulatory effect on PMBCs. Particularly, symptoms of bread made from E82 were evaluated in comparison with gluten-free bread in NCGS patients, showing no differences in the appearance of symptoms [43], meaning that the higher NGP content does not trigger symptoms in NCGS patients. Line E82 is not completely devoid of gluten, and further refining RNAi technology to completely silence additional gluten genes should be required.

The RNAi lines described in this work are of high value for assessing further evaluations in relation to other wheat-related pathologies and intolerances, as some of the NGPs that appear up-regulated play an important role in allergies [34]. In fact, the analysis of the PCA shows that ATIs are not related to the proliferation of PBMCs, nor are any of the other NGPs studied. Other authors have suggested that CD is associated with a robust humoral response directed at a specific subset of unbound gluten proteins, especially serpins [34,44]. Wheat serpins are serine protease inhibitors, and most of them act as a suicide substrate for the inhibition of chymotrypsin-like proteases. Some wheat serpins have been reported that have a recognition region similar to prolamin sequences and the possible prolamin protective function that has been speculated [45]. However, our results do not support a role of serpins increasing immunogenicity.

## 5. Conclusions

The down-regulation of prolamin fractions by RNAi provided wheat lines differing in protein composition and in the content of CD immunogenic epitopes. These RNAi lines showed a reduction in total gliadin content, in specific gliadin fractions (particularly α-gliadins for some lines), and an increment in the HMW fraction, and in NGPs such as ATIs, serpins and triticins. Proliferation assay and INF-γ release revealed three wheat RNAi lines—the stimulatory response of which does not differ from the rice protein extract used as the gluten-free negative control. These lines, especially line E82, present a very low content of gluten as determined by G12, with a pronounced decrease in α-gliadin, containing very low or no DQ2.5 and p31–43 α-gliadin epitopes and with differences in glutenins and different increments in the NGP fraction. The non-gluten protein seems not to play a key role in PBMC proliferation and INF-γ release.

Our work highlights the utility of PBMCs as a highly useful tool for the validation of the stimulatory capacity of wheat lines differing in protein composition triggering immune response in CD.

## Figures and Tables

**Table 1:** RNAi lines obtained from the wild-type BW208 and their corresponding transformation vectors and prolamin silencing targets.

Line	Plasmid 1	Plasmid 2	Prolamin Target
BW208	NA ^1^	NA ^1^	NA ^1^
D623	pghpg8.1	NA ^1^	γ-gliadin
D783	pDhp_ω/α	NA ^1^	ω-, α-, and γ-gliadin
E82	pghpg8.1	pDhp_ω/α	ω-, α-, and γ-gliadin
H320	pDhp_α/βZR	pDhp_ω4ZR	ω- and α-gliadin
H754	pDhp_ω8ZR	NA ^1^	ω-gliadin
H811	pDhp_α/βZR	pDhp_ω8ZR	LMW, ω- and α-gliadin
I17	pDhp_α/βZR	NA ^1^	α-gliadin

^1^ NA, non-applicable; BW208, bread wheat cv. Bobwhite; LMW, low-molecular-weight glutenin subunits.

## Supplementary Materials

The following are available online at https://www.mdpi.com/2072-6643/11/12/2933/s1. Table S1: Epitope list; Table S2: Clinical data of patients with celiac disease; Table S3: SI multiple range tests, Table S4: INF-γ multiple range tests, Table S5: Summary of data from RP-HPLC, LC-MS/MS, G12 moAb and PBMCs.
